# Gene selection for cancer identification: a decision tree model empowered by particle swarm optimization algorithm

**DOI:** 10.1186/1471-2105-15-49

**Published:** 2014-02-20

**Authors:** Kun-Huang Chen, Kung-Jeng Wang, Min-Lung Tsai, Kung-Min Wang, Angelia Melani Adrian, Wei-Chung Cheng, Tzu-Sen Yang, Nai-Chia Teng, Kuo-Pin Tan, Ku-Shang Chang

**Affiliations:** 1 Department of Industrial Management, National Taiwan University of Science and Technology, Taipei 106, Taiwan, R.O.C; 2 Department of Food Science, Yuanpei University, No. 306, Yuanpei Street, Hsinchu 300, Taiwan, R.O.C; 3 Department of Surgery, Shin-Kong Wu Ho-Su Memorial Hospital, Taipei, Taiwan, R.O.C; 4 Pediatric Neurosurgery, Department of Surgery, Cheng Hsin General Hospital, Taipei 11220, Taiwan, R.O.C; 5 Genomic Research Center, National Yang-Ming University, Taipei 11221, Taiwan, R.O.C; 6 School of Dental Technology, Taipei Medical University, Taipei 110, Taiwan, R.O.C; 7 Taiwan Research Center for Biomedical Implants and Microsurgery Devices, Taipei Medical University Taipei 110, Taiwan, R.O.C; 8 School of Dentistry, College of Oral Medicine, Taipei Medical University, Taipei, Taiwan, R.O.C; 9 MBA, School of Management, National Taiwan University of Science and Technology, Taipei 106, Taiwan, R.O.C

**Keywords:** Gene expression, Cancer, Particle swarm optimization, Decision tree classifier

## Abstract

**Background:**

In the application of microarray data, how to select a small number of informative genes from thousands of genes that may contribute to the occurrence of cancers is an important issue. Many researchers use various computational intelligence methods to analyzed gene expression data.

**Results:**

To achieve efficient gene selection from thousands of candidate genes that can contribute in identifying cancers, this study aims at developing a novel method utilizing particle swarm optimization combined with a decision tree as the classifier. This study also compares the performance of our proposed method with other well-known benchmark classification methods (support vector machine, self-organizing map, back propagation neural network, C4.5 decision tree, Naive Bayes, CART decision tree, and artificial immune recognition system) and conducts experiments on 11 gene expression cancer datasets.

**Conclusion:**

Based on statistical analysis, our proposed method outperforms other popular classifiers for all test datasets, and is compatible to SVM for certain specific datasets. Further, the housekeeping genes with various expression patterns and tissue-specific genes are identified. These genes provide a high discrimination power on cancer classification.

## Background

Researchers have tried to analyze thousands of genes simultaneously by microarray technology to obtain important information about specific cellular functions of gene(s) which can be used in cancer diagnosis and prognosis [1]. The gene selection from gene expression data are challenging due to the properties of small sample size, high dimension and high noise. A method is needed for choosing the important subset of genes with high classification accuracy. Such method would not only enable doctors to identify a small subset of biologically relevant genes for cancers, but will also save computational costs [2].

The gene selection method can be divided into three classes, the wrapper, the filter, and the embedded approaches. Wrappers utilize learning machine and search for the best features in the space of all feature subsets. Despite their simplicity and often having the best performance results, wrappers highly depend on the inductive principle of the learning model and may suffer from excessive computational complexity because the learning machine has to be retrained for each feature subset considered [3]. The wrapper method is usually superior to the filter one since it involves intercorrelation of individual genes in a multivariate manner, and can automatically determine the optimal number of feature genes for a particular classifier. The filter approach usually employs statistical methods to collect the intrinsic characteristics of genes in discriminating the targeted phenotype class, such as statistical tests, Wilcoxon’s rank test and mutual information, to directly select feature genes [4]. This approach is easily implemented, but ignores the complex interaction among genes. Finally, the embedded method is a catch-all group technique which performs feature selection as part of the model construction process. It is similar to the wrapper method, while multiple algorithms can be combined in the embedded method to perform feature subset selection [5,6]. Genetic algorithms (GAs) [7] are generally used as the search engine for feature subset in the embedded method, while other classification methods, such as estimation of distribution algorithm (EDA) with SVM [8-13], K nearest neighbors/genetic algorithms (KNN/GA) [14], genetic algorithms-support vector machine (GA-SVM) [15] and so forth, are used to select feature subset.

Particle Swarm Optimization (PSO), developed by Kennedy and Eberhart [16], is a population-based meta-heuristic on the basis of stochastic optimization, inspired by the social behavior of flocks of birds or schools of fish [17]. PSO has been widely applied in many fields to solve various optimization problems, including gene selection [1,2,18-20]. A swarm of particles with randomly initialized positions would move toward the optimal position along the search path that is iteratively updated on the basis of the best particle position and velocity in PSO. The potential solutions, called particles, are used to represent a candidate solution for the problem. Among the classifiers given a specific search algorithm, C4.5 is a decision tree-based classifier listed in the top 10 most influential data-mining algorithms [21]. Decision trees are a linear method which is easy to interpret and understand.

This paper presents a PSO-based algorithm to address the problem of gene selection. The proposed approach is an integration of PSO searching algorithm and C4.5 decision tree classifier, called PSODT. Combining PSO with C4.5 classifier has rarely been investigated by previous researchers. The performance of our proposed method will be evaluated by 11 microarray datasets, which consist of 1 dataset from cancer patients of the M^2^ DB in Taiwan [22] and 10 from the Gene Expression Model Selector [23]. In addition, the performance of our proposed method will be compared with other well-known classifier algorithms, such as self-organizing map (SOM), C4.5, back propagation neural network (BPNN), SVM, NaivaBayes (NB), CART decision tree, and artificial immune recognition system (AIRS). Statistical test will be employed to discriminate the difference of all the algorithms in terms of classification accuracy.

### Gene selection and classification

DNA microarray (also commonly known as DNA chip or biochip) is a collection of microscopic DNA spots attached to a solid surface and allows researchers to measure the expression levels of thousands of genes simultaneously in a single experiment. The DNA microarray is operated by classifier approaches to compare the gene expression levels in tissues under different conditions [24]; for instance, the study of Jiang et al. [25] devised an RF-based method to classify real pre-miRNAs using a hybrid feature set for the wild type versus mutant, or healthy versus diseased classes. Batuwita and Palade [26] developed a classifier named micro-Pred for distinguishing human pre-miRNA hairpins from both pseudo hairpins and other ncRNAs. Wang et al. [27] presented a hybrid method combining GA and SVM to identify the optimal subset of microarray datasets, and claimed their method was superior to those obtained by microPred and miPred. Further, Nanni et al [28] recently devised a support vector machine (SVM) as classifier for microarray gene classification. Their method combines different feature reduction approaches to improve classification performance of the accuracy and area under the receiver operating characteristic (ROC). Park et al [29] presented a method for inferring combinatorial Boolean rules of gene sets for cancer classification and cancer transcriptome. Their study identified a small group of gene sets that synergistically contribute to the classification of samples into their corresponding phenotypic groups (such as normal and cancer) and reduced the search space of the possible Boolean rules.

Due to the high computational cost and memory usage for classifying high dimensional data, appropriate gene selection procedure is required to improve classification performance. As addressed by Tan et al. [30], given the quantity and complexity of the gene expression data, it is unlikely to efficiently compute and compare the n × m gene expression matrix by manually. Instead, machine learning and other artificial intelligence techniques have potential to characterize gene expression data promptly [8,31,32].

### Previous study

Some studies have proposed PSO algorithm for gene selection problems. For instance, Alba et al. [1] presented a modified PSO (geometric PSO) for high-dimensional microarray data. Both augmented SVM and GA were proposed for comparison on six public cancer datasets. Li et al. [23] devised a method of combining PSO with a GA and adopted SVM as the classifier for gene selection. Their proposed approach used three benchmark gene expression datasets for validation: leukemia, colon cancer, and breast cancer. Mohamad et al. [19] presented an improved binary PSO combined with an SVM classifier to select a near-optimal subset of informative genes relevant to cancer classification.

Zhao et al. [33] lately presented a novel hybrid framework (NHF) for gene selection and cancer classification of high dimensional microarray data by combining the information gain (IG), F-score, GA, PSO, and SVM. Their method was compared to PSO-based, GA-based, ant colony optimization-based, and simulated annealing (SA)-based methods on five benchmark data sets: leukemia, lung carcinoma, colon, breast, and brain cancers. Chen et al. [18] used PSO + 1NN for feature selection and tested their algorithm against 8 benchmark datasets from UC Irvine Machine Learning Repository as well as to a real case of obstructive sleep apnea. Previous research all indicates that PSO is promising to solve the gene selection problem.

## Methods

We integrated PSO algorithm with the C4.5 classifier to address the gene selection problem (refer to Appendix 1 & 2 at [34]). The important genes were proposed using PSO algorithm, and then C4.5 was employed as a fitness function of the PSO algorithm to verify the efficiency of the selected genes.

### Solution/particle representation and initialization

A particle represents a potential solution (i.e., gene subset) in an *n*-dimensional space. The particles used binary digits string with length *n*, the total number of genes for gene selection. The bits consisted of 0 and 1 digits, which correspond to non-selected and selected gene, respectively. Each particle was coded as binary alphabetical string. For instance, a particle of ‘11000’ contains five genes where only the first and the second gene were selected. We updated the dimension *d* of particle *i* by $x_{\mathrm{id}}^{\mathrm{new}}=\left\{\begin{array}{c} 1,\;\mathrm{if}\;\mathrm{sigmoid}\left(v_{\mathrm{id}}^{\mathrm{new}}\right)>U\left(0,1\right)\\ 0,\;\mathrm{otherwise} \end{array}\right)\;\mathrm{where}\;\mathrm{the}\;\mathrm{sigmoid}\left(v_{\mathrm{id}}^{\mathrm{new}}\right)\;\mathrm{is}\;1/\left(1+e^{-v_{\mathrm{id}}^{\mathrm{new}}}\right)$.

We used a random function to initialize the particle population of PSO. Seeding PSO with a good initial can lead to a better result. This study has examined two generators of random seeds to initiate solutions: the first is generated by using Visual C# random seed function and the second is from a uniform distribution with a range from 0 to 1, denoted as of *U*(0,1). The result (as shown in Table 1) reveals that *U*(0,1) outperforms Visual C# random seed generator. In this study, a probability of 0.5 is randomly assigned to bit values 0 and 1. If U(0,1)>0.5, then $x_{\mathrm{id}}^{0}=1$; otherwise, $x_{\mathrm{id}}^{0}=0$.

**Table 1:** Random seed comparison

		**Visual C#**	***U*** **(0,1)**
11_Tumors	1	96.22	97.52
	2	95.31	98.32
	3	96.72	98.04
	4	96.31	97.66
	5	97.62	97.57
	Avg.	96.44	97.82
	(Std.)	0.84	(0.35)
DLBCL	1	91.31	91.88
	2	89.70	92.22
	3	88.77	91,99
	4	88.72	92.87
	5	92.99	93.22
	Avg.	90.30	92.55
	(Std.)	1.83	(0.61)
14_Tumors	1	65.00	74.00
	2	63.00	75.00
	3	63.00	75.00
	4	63.00	74.00
	5	67.00	75.00
	Avg.	64.20	74.60
	(Std.)	1.79	(0.55)

### Fitness function and PSO procedure

**Figure 1:**
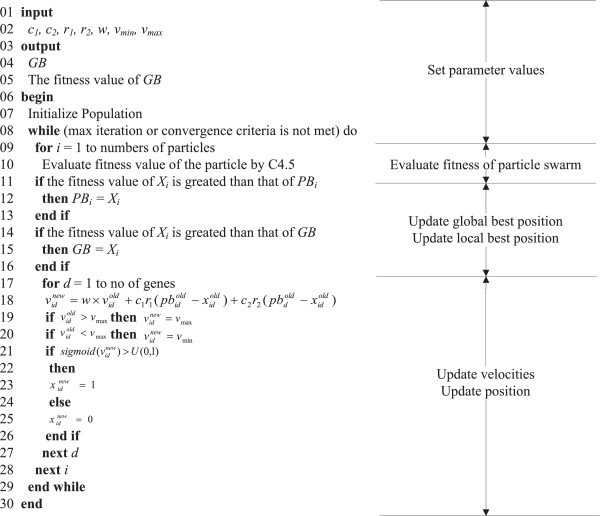
The proposed PSODT for gene selection.

The PSO fitness function is based on the classification accuracy measured by the C4.5 classifier. Figure 1 shows the procedure of applying PSODT on gene selection.

## Results and discussion

### Experimental setting

This study used 10 microarray cancer datasets (with diverse sizes, features, and classes) and conducted numerical experiments to evaluate the performance of our proposed method. The 10 datasets were obtained from GEMS [23], including 11_Tumors, 14_Tumors, 9_Tumors, Brain_Tumor1, Brain_Tumor2, Leukemia2, Lung_Cancer, SRBCT, Prostate_Tumor, and DLBCL. In GEMS dataset, these types of cancer belong in the top 10 in terms of cancer incidences and deaths in USA in 2012. Table 2 summarizes the characteristics of those microarray datasets. In addition, five sets of cDNA clones were selected and used individually for this purpose (refer to [34]).

**Table 2:** Microarray datasets employed in the study

**Data set**	**Feature size**	**Sample size**	**Class size**	**Particle size**
11_Tumors	12601	203	5	126
14_Tumors	10368	50	4	104
9_Tumors	10368	50	4	104
Brain_Tumor1	5727	60	9	57
Brain_Tumor2	12534	174	11	125
Leukemia2	5328	72	3	53
Lung_Cancer	11226	72	3	112
SRBCT	5470	77	2	55
Prostate_Tumor	10510	102	2	105
DLBCL	83	2309	4	10

The PSO parameters are chosen by a survey on several related research articles concerning the utilization of PSO. Such parameter setting was optimized by literatures (refer to [35-37]). Moreover, we conducted many trials to test such parameter setting which shows the best objective value. The parameters used for PSODT are as follows. The number of particles in the population was set to the one-tenth number of genes (features) (refer to the field of ‘particle size” in Table 2). The parameter, *c*_*1*_ and *c*_*2*_, were both set at 2, whereas the parameter, lower (*v*_*min*_) and upper bounds (*v*_*max*_), were set at -4 and 4, respectively. The inertia weight (*w*) was set at 0.4. Random factors, *r*_*1*_ and *r*_*2*_, are within [0, 1] interval. The process was repeated until either the fitness of the given particle was 1.0 or the number of the iterations was achieved by the default value of *T* = 100. Table 2 shows the summarization of microarray dataset characteristics.

### Cross-validation

To guarantee the impartial comparison of the classification results and avoid generating random results, this study adopted a five-fold cross-validation strategy. Cross-validation is a statistical method by dividing data into two segments for evaluating and comparing learning algorithms. One part used to learn or train a model and the other used to validate the model. Stone [38] and Geisser [39] employed cross-validation as means for choosing proper model parameters, as opposed to using cross-validation purely for estimating model performance [40-42]. *K*-fold cross-validation is used to evaluate algorithms. In this study we set *K* = 5, and the details are stated as follows: in each iteration, the algorithms apply *K* folds of data to earn one or more models, and subsequently the learned models are asked to predict the data in the validation fold. The performance of the algorithm on each fold is tracked by its accuracy. Upon completion, the *K* samples of the accuracy is available for validation.

### An illustration of the resulting cancer classifier structure

**Figure 2:**
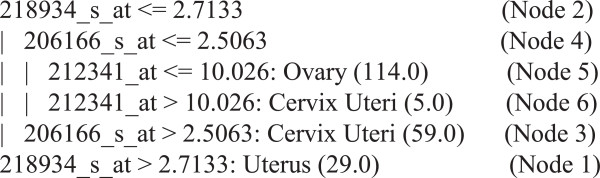
An illustration of partial decision tree.

Figure 2 demonstrates a sample decision tree for classifying three female cancers (i.e., ovary, cervix uteri and uterus). The genes causing cancers led to a classification tree with four terminal nodes (or clusters of cancer). For instance, 218934_s_at, 206166_s_at and 212341_at are identified as splitters. 218934_s_at are strongly associated with the three cancers; the first branch of the tree is based on 218934_s_at: a high score (i.e., 218934_s_at > 2.7133) implies the occurrence of uterus cancer (Node 1). When 218934_s_at < = 2.7133 (Node 2), 206166_s_at > 2.5063 implies the occurrence of cervix uteri cancer (Node 3), and when 206166_s_at < = 2.5063 (Node 4) and 212341_at < = 10.026, it implies the occurrence of ovary cancer (Node 5); otherwise, 212341_at > 10.026 implies again the occurrence of cervix uteri cancer (Node 6).

### Benchmark results with other classification algorithms

**Figure 3:**
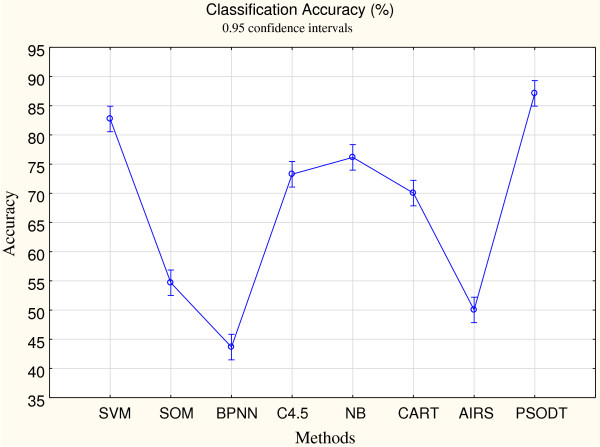
95% confidence interval of the mean for classification accuracy.

To confirm effectiveness of our proposed PSODT, this study compares its accuracy with the other seven popular classification algorithms (i.e., SVM, SOM, BPNN, C4.5, BN, CART, and AIRS). Table 3 shows the accuracy of our proposed method as compared to the other four algorithms. Five-fold cross-validation is applied on the datasets and the average and standard deviations were obtained. Our proposed method was superior to the others, except it is compatible to SVM for two datasets, 9_Tumors and SRBCT. The stability (convergence) shows that the standard deviation of PSODT is less than 1%. Figure 3 shows the averaged classification accuracy in 95% confidence interval (with respect to the 10 datasets) which indicates that PSODT outperformed the other algorithms. This study used two-way ANOVA to determine whether the eight algorithms were significantly different in terms of average classification accuracy. The result fulfills the ANOVA assumptions on normality, homoscedasticity and independence. In ANOVA analysis, the classification algorithms defined as “factor”, whereas the datasets were defined as “block”. Table 4 lists the ANOVA results for average classification accuracy. The results showed significant differences of classification accuracy among the 8 algorithms. Further, to determine if each pair of the five algorithms differed from each other, Fisher’s test was used in this study, as shown in Table 5. The p-values demonstrate that our proposed method exhibits differences in mean classification accuracy as compared with the other algorithms, except it is compatible with SVM. Table 6 shows the computational time for each algorithm. Although the time consumed by the proposed tree based algorithm is significantly larger than the others, it is within a reasonable range even for the large-sized datasets.

**Table 3:** Classification accuracy for 10 microarray datasets (%)

**Data set**	**Run no.**	**SVM**	**SOM**	**BPNN**	**C4.5**	**NB**	**CART**	**AIRS**	**PSODT**
11_Tumors	1	93.85	78.76	69.38	90.31	80.3	84.73	59.61	97.52
	2	93.79	79.22	69.12	91.22	80.79	85.22	59.61	98.32
	3	94.22	78.98	69.74	90.87	80.79	84.73	56.65	98.04
	4	94.01	77.22	68.99	89.81	80.47	86.21	56.65	97.66
	5	93.60	77,81	69.21	88.72	80.30	84.73	60.59	97.57
	Avg.	93.89	78.55	69.29	90.19	80.53	85.12	58.62	97.82
	(Std.)	(0.23)	(0.90)	(0.29)	(0.98)	(0.25)	(0.64)	(1.84)	(0.35)
14_Tumors	1	60.00	40.00	28.00	48.00	72.00	56.00	54.00	74.00
	2	62.00	40.00	30.00	48.00	70.00	54.00	54.00	75.00
	3	60.00	40.00	28.00	48.00	72.00	54.00	52.00	75.00
	4	60.00	40.00	28.00	48.00	74.00	56.00	52.00	74.00
	5	62.00	40.00	29.00	48.00	70.00	54.00	54.00	75.00
	Avg.	60.80	40.00	28.60	48.00	71.60	54.80	53.20	74.60
	(Std.)	(1.10)	(0.00)	(0.89)	(0.00)	(1.67)	(1.10)	(1.10)	(0.55)
9_Tumors	1	76.00	40.00	34.00	52.00	70.00	62.00	48.00	74.00
	2	76.00	40.00	34.00	52.00	70.00	62.00	46.00	74.00
	3	76.00	40.00	34.00	52.00	70.00	62.00	46.00	74.00
	4	76.00	40.00	34.00	52.00	70.00	64.00	46.00	74.00
	5	76.00	40.00	34.00	52.00	70.00	60.00	46.00	74.00
	Avg.	76.00	40.00	34.00	52.00	70.00	62.00	46.40	74.00
	(Std.)	(0.00)	(0.00)	(0.00)	(0.00)	(0.00)	(1.41)	(0.89)	(0.00)
Brain_Tumor1	1	49.20	18.33	15.00	41.67	41.67	30.00	33.33	56.34
	2	49.71	19.72	15.21	41.89	41.33	28.33	35.00	57.22
	3	49.06	18.34	15.30	42.00	41.67	30.00	31.67	57.31
	4	48.78	17.78	15.23	41.79	41.67	33.34	30.00	56.96
	5	49.22	18.40	15.00	41.98	40.67	30.00	35.00	57.33
	Avg.	49.19	18.51	15.15	41.87	41.40	30.33	33.00	57.03
	(Std.)	(0.34)	(0.72)	(0.14)	(0.14)	(0.43)	(1.83)	(2.17)	(0.41)
Brain_Tumor2	1	83.03	50.00	25.28	74.12	81.61	71.24	48.28	85.75
	2	83.77	48.21	25.22	73.98	85.06	71.84	47.7	86.22
	3	83.21	49.77	25.28	74.22	84.48	70.11	44.83	86.52
	4	83.70	49.21	25.31	74.29	81.61	71.24	45.98	86.13
	5	84.01	49.80	25.30	74.22	83.33	69.54	48.28	85.70
	Avg.	83.54	49.40	25.28	74.17	83.22	70.79	47.01	86.06
	(Std.)	(0.41)	(0.73)	(0.03)	(0.12)	(1.59)	(0.94)	(1.54)	(0.34)
Leukemia2	1	93.89	60.86	50.10	90.29	93.06	84.72	47.22	100.00
	2	94.22	60.73	50.01	90.33	93.06	84.72	44.44	100.00
	3	93.71	60.80	50.22	90.21	94.44	83.33	48.61	100.00
	4	93.72	60.78	50.19	90.76	93.06	84.72	45.67	100.00
	5	93.80	62.09	50.31	90.52	94.44	81.94	47.22	100.00
	Avg.	93.87	61.05	50.17	90.42	93.61	83.89	46.63	100.00
	(Std.)	(0.21)	(0.58)	(0.12)	(0.22)	(0.76)	(1.24)	(1.61)	(0.00)
Lung_Cancer	1	98.27	68.19	39.14	90.29	93.06	73.61	57.72	100.00
	2	98.22	68.27	39.24	90.31	93.06	73.61	55.78	100.00
	3	97.43	67.85	39.17	90.22	93.06	73.61	57.72	100.00
	4	97.47	68.33	38.79	90.54	91.67	73.61	55.56	100.00
	5	97.12	68.12	39.01	90.31	93.06	73.61	57.72	100.00
	Avg.	97.70	68.15	39.07	90.33	92.78	73.61	56.90	100.00
	(Std.)	(0.51)	(0.19)	(0.18)	(0.12)	(0.62)	(0.00)	(1.13)	(0.00)
SRBCT	1	96.62	67.67	83.08	78.00	80.52	87.01	51.95	92.49
	2	95.42	68.07	83.19	78.66	80.52	85.12	53.64	93.21
	3	96.01	68.31	82.86	78.21	79.22	85.42	53.05	93.08
	4	95.55	67.90	82.99	77.97	81.82	87.01	53.24	92.82
	5	96.01	67.21	83.01	77.90	81.82	88.32	51.95	93.10
	Avg.	95.92	67.83	83.03	78.15	80.78	86.58	52.77	92.94
5	(Std.)	(0.47)	(0.42)	(0.12)	(0.31)	1.09	1.31	0.77	(0.29)
Prostate_Tumor	1	88.04	66.71	56.62	88.14	62.75	82.35	51.96	94.10
	2	87.91	67.51	56.77	88.14	62.75	83.33	52.94	94.64
	3	87.11	66.88	56.49	88.18	61.76	82.35	52.94	94.21
	4	88.23	67.02	56.80	88.14	62.75	82.35	52.75	94.49
	5	87.03	68.07	56.92	88.14	61.76	82.35	51.96	94.11
	Avg.	87.66	67.24	56.72	88.15	62.35	82.55	52.51	94.31
	(Std.)	(0.55)	(0.55)	(0.17)	(0.02)	0.54	0.44	0.51	(0.24)
DLBCL	1	88.42	51.47	35.88	79.34	85.11	73.49	50.6	91.88
	2	88.71	52.28	34.71	79.21	86.75	69.88	53.01	92.22
	3	89.09	51.77	34.98	79.44	85.11	69.88	54.93	91,99
	4	88.68	51.29	35.06	79.83	84.34	69.88	53.37	92.87
	5	88.97	51.49	35.78	79.20	85.11	69.88	54.21	93.22
	Avg.	88.77	51.66	35.28	79.40	85.28	70.60	53.22	92.55
	(Std.)	(0.26)	(0.39)	(0.52)	(0.26)	0.88	1.61	1.65	(0.61)

**Table 4:** ANOVA for average classification accuracy

	**SS**	**DF**	**MS**	**F**	**P**
Method	88638	7	12662.61	2.254880	0.029801
Dataset	76054	9	8450.40	1.504796	0.144956
Method × Dataset	22367	63	355.04	0.063223	1.000000
Error	1802622	321	5615.65		

**Table 5:** ***p*-value of multiple comparison for average classification accuracy**

	**SVM**	**SOM**	**BPNN**	**C4.5**	**NB**	**CART**	**AIRS**	**PSODT**
SVM		0.000000	0.000000	0.000000	0.000035	0.000000	0.000000	0.005543
SOM	0.000000		0.000000	0.000000	0.000000	0.000000	0.003209	0.000000
BPNN	0.000000	0.000000		0.000000	0.000000	0.000000	0.000061	0.000000
C4.5	0.000000	0.000000	0.000000		0.066845	0.039928	0.000000	0.000000
NB	0.000035	0.000000	0.000000	0.066845		0.000114	0.000000	0.000000
CART	0.000000	0.000000	0.000000	0.039928	0.000114		0.000000	0.000000
AIRS	0.000000	0.003209	0.000061	0.000000	0.000000	0.000000		0.000000
PSODT	0.005543	0.000000	0.000000	0.000000	0.000000	0.000000	0.000000	

**Table 6:** CPU time (in sec.)

**Data set**	**SVM**	**SOM**	**BPNN**	**C4.5**	**NB**	**CART**	**AIRS**	**PSODT**
11_Tumors	6.63	33.57	252.10	27.53	36.21	110.75	50.90	1203.80
14_Tumors	0.63	3.90	31.16	2.06	5.70	10.62	14.12	139.53
9_Tumors	0.63	4.57	36.08	3.21	5.21	14.21	15.69	164.05
Brain_Tumor1	1.17	5.24	42.14	3.93	5.73	17.66	10.37	186.45
Brain_Tumor2	8.35	38.11	295.33	35.49	32.02	160.34	45.73	1348.20
Leukemia2	0.53	2.86	23.73	1.43	5.51	8.33	20.51	101.29
Lung_Cancer	0.98	5.64	44.21	3.13	9.12	19.88	21.00	198.62
SRBCT	0.31	2.77	21.23	1.37	5.52	8.53	14.99	99.10
Prostate_Tumor	1.12	8.37	64.40	2.87	13.08	18.34	34.66	300.58
DLBCL	0.30	1.64	13.66	1.11	3.22	5.90	15.21	58.06

In summary, SVM classification method which is generally considered as one of the most powerful machine learning classifiers is based on the statistical learning theory [43]. However, the structure of SVM is a black box system which does not provide insights on the reasons of a classification or explanations similar to ANN. SOM is one of the categories of ANN algorithms for supervised learning. BPNN is a common type of ANN and capable to recognize complex patterns in data. However, all these abovementioned classifiers are black box systems and nonlinear models. NB classifier considers each of these features to contribute independently to the probability, regardless of the presence or absence of the other features. CART may be no good binary split on an attribute that has a good multi-way split [44], which may lead to inferior trees. AIRS have many parameters that is not easy to find the optimum combination of parameters. Instead, C4.5 is a classifier that creates a decision tree based on rules, and is a linearly method simple to understand and interpret. This study integrates the nonlinear search capability of PSO and linearly separable advantage of DT.

### Model justification by a clinical dataset

This study investigated a set of clinical practice data including 13 actual cancer cases from the M^2^ data bank in Taiwan [22]. The raw intensity data of cancer (CEL files) generated using Affymetrix HG-U133A and HG-U133 plus 2.0 platforms were retrieved from Array Express and Gene expression omnibus (GEO). Arrays performed with samples other than human clinical specimens, such as cell lines, primary cells, and transformed cells, were excluded.

All raw data of microarray (5,335 samples) were pre-processed using three different algorithms: Affymetrix Microarray Suite 5 (MAS5), robust multi-chip average (RMA), and GC-robust multi-chip average (GCRMA) as implemented in the Bioconductor packages. RMA and GCRMA processed data on a multi-array basis. All of the arrays of the same platform were uniformly pre-processed to reduce variance. The cancer microarray consisted of 13 cancer types, namely, bladder, blood, bone marrow, brain, breast, cervix uterus, colon, kidney, liver, lung, lymph node, ovary, and prostate. The information of each cancer is shown in Table 7.

**Table 7:** The arrays of cancers

**#**	**Cancer**	**Sample size**
1	Bladder	94
2	Liver	107
3	Cervix Uteri	148
4	Prostate	166
5	Lung	222
6	Brain	250
7	Lymph Node	280
8	Kidney	301
9	Ovary	331
10	Colon	437
11	Blood	503
12	Bone Marrow	676
13	Breast	1817

Table 8 presents the classification accuracy of PSODT for each run and the number of genes selected. The accuracy of PSODT and SVM were 97.26 and 72.46, respectively. The test results on the 13 cancer microarrays for all benchmark algorithms are shown in Table 9. The results indicated that PSODT outperformed the SVM and other benchmark methods.

**Table 8:** Classification accuracies for each run using PSODT

**Data set**	**Run no.**	**Classification accuracy (%)**	**No. selected genes**
13 cancer	1	97.26	135
	2	98.72	100
	3	97.25	134
	4	97.79	135
	5	97.39	126
	Avg.	97.68	126
	(Std.)	(0.62)	(15.02)

**Table 9:** Classification accuracy for 13 sets of cancer microarray (%)

**Data set**	**Run no.**	**SVM**	**SOM**	**BPNN**	**C4.5**	**NB**	**CART**	**AIRS**	**PSODT**
13 cancer	1	72.46	52.60	42.58	93.14	94.21	91.42	50.41	97.26
	2	72.46	52.77	41.77	93.25	93.78	90.54	52.31	98.72
	3	72.51	51.39	42.59	93.26	93.60	91.77	53.33	97.25
	4	72.51	52.60	42.33	93.25	94.81	92.04	53.85	97.79
	5	73.62	52.60	43.33	93.25	94.02	91.09	53.71	97.39
	Avg.	72.71	52.39	42.52	93.23	94.08	91.37	52.72	97.68
	(Std.)	(0.51)	(0.56)	(0.56)	(0.05)	(0.47)	(0.59)	(1.43)	(0.62)

To perform a five-fold cross-validation, we selected five independent sets of cDNA clones (refer to supplementary Tables one to five of Appendix three at [34]). A total of 453 cDNA clones were selected at least once. Among the lists of cDNA clones, a number of them were selected multiple times. The genes being selected multiple times (with Frequency ≥ 4) indicate that the expression levels of these genes provide a high discrimination power among the tumors of different anatomical origin. Therefore, these genes are likely to be the tissue-specific genes. Alternatively, such expression differences may be generated result from organ- or tissue-specific malignant transformation.

## Conclusions

We proposed a novel method to identify tissue-specific genes as well as housekeeping genes with altered expression patterns that provide a high discrimination power on cancer classification. These genes may play as an important role in diagnosis and/or pathogenesis of various types of tumors. Eleven cancer datasets were used to test the performance of the proposed method, and a five-fold cross-validation method was used to justify the performance of our proposed method. Our proposed approach achieved a higher accuracy as compared with all the other methods.

This proposed method has integrated with the nonlinear search capability of PSO and linearly separable advantage of DT to apply to microarray cancer datasets for gene selection. Hawse have identified representative cancer genes (453 genes) from numerous microarray data (65,000 genes) that can reduce costs. In addition, we compared our proposed method with four well-known algorithms using a variety of datasets (diverse sizes and numbers of classes and features). Consequently, our proposed method outperformed all the other benchmark methods and is compatible to SVM for certain specific datasets.

Further studies to be further conducted are suggested as follows. First, PSO may result in better solutions by optimizing parameter settings; therefore, self-adaptation parameters of particle size, number of iterations, and constant weight factors are worth developing. Second, adding hybrid search algorithms in PSO algorithm may improve its performance; for example, swarms with mixed particles may further enhance the effectiveness. Third, the improvement in the execution time for large-sized data sets could be treated as a research subject in the future.

## Competing interests

The authors declare that they have no competing interests.

## Authors’ contributions

KHC, KJW and AMA designed all the experiments and wrote most of this paper. KSC discussed and refined the paper. MLT, WCC, KPT and KMW interpreted the results. TSY and NCT guided the whole project. All authors read and approved the final manuscript.
